# Human respiratory syncytial virus and influenza seasonality patterns—Early findings from the WHO global respiratory syncytial virus surveillance

**DOI:** 10.1111/irv.12726

**Published:** 2020-03-12

**Authors:** Mandeep Chadha, Siddhivinayak Hirve, Christina Bancej, Ian Barr, Elsa Baumeister, Braulia Caetano, Malinee Chittaganpitch, Badarch Darmaa, Joanna Ellis, Rodrigo Fasce, Herve Kadjo, Sandra Jackson, Vivian Leung, Maria Pisareva, Jocelyn Moyes, Amel Naguib, Almiro Tivane, Wenqing Zhang, the WHO RSV Surveillance Group, Amal Barakat, Sumit Bhardwaj, Shobha Broor, Alyeksandr Burmaa, Harry Campbell, Daouda Coulibaly, Nigel Crawford, Manal Fahim, Belinda Herring, Harish Nair, Rakhee Palekar, Richard Pebody, Soatiana Rajatonirina, Marilda Siqueira, Peter G. Smith, Elizaveta Smorodintseva, Viviana Sotomayor, Florette Treurnicht, Miquelina Vaz, Marietjie Venter, Niteen Wairagkar, Maria Zambon

**Affiliations:** ^1^ National Institute of Virology Indian Council of Medical Research Pune India; ^2^ Global Influenza Programme World Health Organization Geneva Switzerland; ^3^ Centre for Immunization and Respiratory Infections Public Health Agency of Canada Ottawa Canada; ^4^ Victorian Infectious Diseases Reference Laboratory Peter Doherty Institute for Infection and Immunity Melbourne Australia; ^5^ Departamento Virologia INEI‐ANLIS “Carlos G Malbrán” Buenos Aires Argentina; ^6^ Institute Oswaldo Cruz/FIOCRUZ Rio de Janerio Brazil; ^7^ Department of Medical Sciences Ministry of Public Health Nonthaburi Thailand; ^8^ Virology Laboratory National Center for Communicable Diseases Ulan baatar Mongolia; ^9^ Virus Reference Department Public Health England London United Kingdom; ^10^ Sub‐department of Viral Diseases Instituto de Salud Pública de Chile Santiago Chile; ^11^ Department of Epidemic Viruses Institut Pasteur de Côte d’Ivoire Abidjan Côte d’Ivoire; ^12^ Laboratory of Molecular Virology Smorodintsev Research Institute of Influenza St. Petersburg Russian Federation; ^13^ Center for Respiratory Diseases and Meningitis National Institute for Communicable Diseases Johannesburg South Africa; ^14^ Central Public Health Laboratory Ministry of Health Cairo Egypt; ^15^ Laboratório de Isolamento Viral Instituto Nacional de Saúde Maputo Mozambique

**Keywords:** Global Influenza Surveillance and Response network, human respiratory syncytial virus, influenza, seasonality

## Abstract

**Background:**

Human respiratory syncytial virus (RSV) causes illnesses among all age groups and presents a burden to healthcare services. To better understand the epidemiology and seasonality of RSV in different geographical areas, the World Health Organization (WHO) coordinated a pilot initiative to access the feasibility of establishing RSV surveillance using the existing Global Influenza Surveillance and Response System (GISRS) platform.

**Objectives:**

To describe and compare RSV and influenza seasonality in countries in the northern andsouthern temperate, and tropics during the period January 2017 to April 2019.

**Methods:**

Fourteen countries in six WHO regions participating in the GISRS were invited for the pilot. Hospitalized patients presenting with severe acute respiratory illness (SARI), SARI without fever and outpatients presenting with acute respiratory illness (ARI) were enrolled from January 2017 to April 2019. The expected minimum sample size was 20 samples per week, year‐round, per country. Real‐time RT‐PCR was used to detect RSV and influenza viruses**.** Results were uploaded to the WHO FluMart platform.

**Results:**

Annual seasonality of RSV was observed in all countries, which overlapped to a large extent with the influenza activity. In countries, in temperate regions RSV peaked in the autumn/winter months. In Egypt, a subtropical country, RSV activity peaked in the cooler season. In the tropical regions, RSV peaked during the rainy seasons.

**Conclusion:**

Early findings from the WHO RSV surveillance pilot based on the GISRS suggest annual seasonal patterns for of RSV circulation that overlap with influenza. RSV surveillance needs to be continued for several more seasons to establish seasonality patterns to inform prevention and control strategies.

## 1 INTRODUCTION

Globally, human respiratory syncytial virus (RSV) is recognized as the most common cause of acute lower respiratory infection (ALRI) in the pediatric population.^1^ ALRIs (bronchiolitis and pneumonia) due to RSV in children are among the most frequent causes of hospital admission. It was estimated that, globally, in 2015, there were 3.2 million (uncertainty range [UR] 2.7‐3.8 million) RSV‐associated hospital admissions and nearly 59 600 (UR 48 000‐74 500) in‐hospital deaths from RSV in young children. The total estimated number of deaths due to RSV among young children in developing countries in 2015 was 118 200 (UR 94 600‐149 400). During the neonatal period, 6.5% (95% CI 5.8‐7.6) RSV infection can present as apnea or sepsis.^2^, ^3^ RSV disease among young children may be associated with long‐term sequelae, including recurrent wheezing and asthma, though whether these associations are causal or due to shared susceptibility is unclear. RSV not only affects children but also causes annual outbreaks of respiratory illnesses among all age groups^4^, ^5^ particularly affecting the elderly and adults with comorbidities such as diabetes, heart and lung disease.^6^, ^7^

Given the high RSV disease burden, the development of effective preventive and therapeutic strategies are of high priority. Currently, 19 RSV vaccine candidates and monoclonal antibodies are in various stages of development as prophylactic interventions.^8^ To formulate appropriate intervention strategies, a standardized global RSV surveillance system is needed to better describe the epidemiological characteristics of RSV disease, including seasonal variations in incidence in different geographic settings. In several high‐income countries, surveillance of RSV has been integrated within their routine influenza surveillance^9^; however, there is a paucity of data from low‐ and middle‐income countries (LMICs), where the RSV disease burden is likely the highest.

The World Health Organization (WHO) Global Influenza Surveillance and Response System (GISRS) has been monitoring influenza viruses for over six decades. GISRS operates through a worldwide network of laboratories that provides real‐time surveillance information on the circulation and evolution of influenza viruses. Most of the countries participating in GISRS use a case definition of severe acute respiratory infection (SARI), influenza‐like illness(ILI), and/or acute respiratory infection (ARI), to identify potential influenza cases for laboratory testing, a surveillance system which could be leveraged to build global RSV surveillance. Following several meetings with influenza and RSV experts from different countries, it was agreed to assess the feasibility of establishing RSV surveillance as a pilot using the existing GISRS platform. The pilot would also assess the possibility of compromise to influenza surveillance resulting from the integration of RSV through GISRS.^10^ One of the primary objectives of the RSV pilot was to analyze the seasonal patterns of RSV disease in different countries in varied geographical regions. Fourteen countries, in six WHO regions, which were already members of GISRS, were selected and invited for the pilot.

This paper aims to describe RSV seasonality in countries participating in the WHO RSV pilot surveillance programme during the period January 2017 to April 2019, compared to the seasonality of influenza. Other aspects of the pilot initiative are being published in separate papers.

## 2 MATERIALS AND METHODS

### 2.1 Study sites

**Figure 1:**
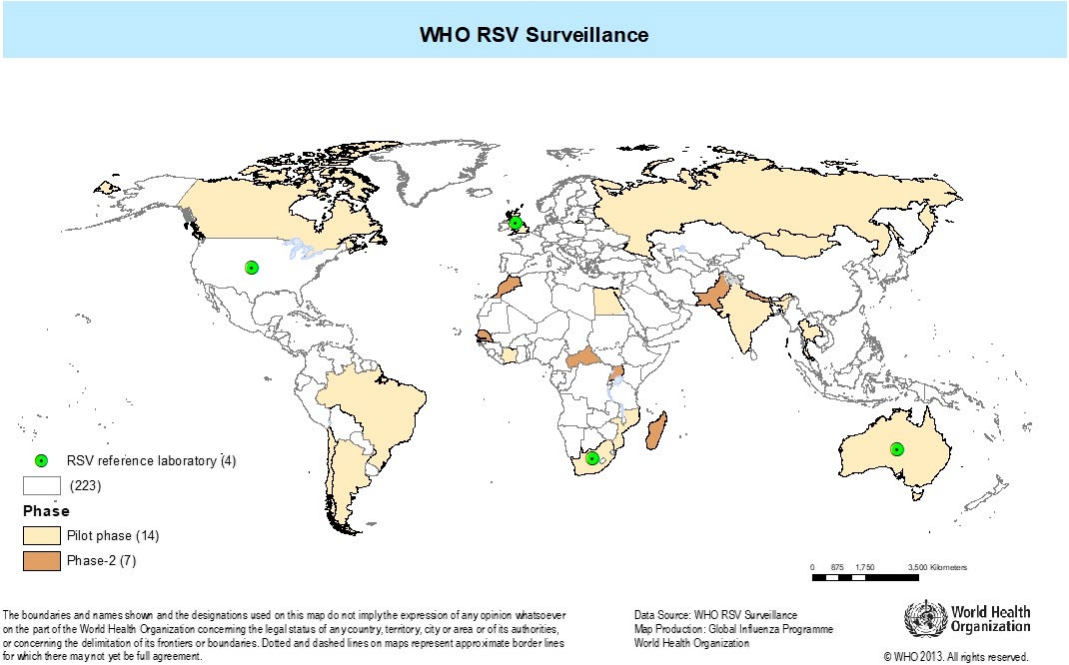
Countries participating in the WHO RSV surveillance

Fourteen countries, in six WHO regions, which were already participating in GISRS, were invited to participate in the pilot (Figure 1). In each of these countries, there was a recognized National Influenza Center and/or a national public health laboratory with ongoing influenza surveillance and laboratory capacity for RSV testing using molecular methods and a history of successful past performance in the WHO EQA for the molecular detection of influenza. All these laboratories/countries regularly provide influenza surveillance data to WHO via the FluNet platform.

### 2.2 Selection of sentinel sites and case definition

The number and type of sentinel hospitals (secondary‐ and tertiary‐level care) and clinics included in the RSV Surveillance Pilot varied among the countries (Table 1). The selection of sentinel hospitals and clinics was largely based on patient load and convenience. These sites were not necessarily nationally representative. For clinic‐ and hospital‐based surveillance, countries chose sentinel sites that were able to comply with the required minimum sample size. Hospitals with inpatient care and intensive care units providing adult and pediatric care were preferentially selected. At these sites, patients across all ages who presented with extended SARI were eligible for inclusion. The SARI case definition requires cough and hospitalization plus the presence, or a history, of fever, whereas the extended SARI definition does not require fever. In addition, infants less than 6 months presenting with sepsis or apnea were also eligible for inclusion, as RSV illness frequently presents with these conditions in this age group.^10^ For outpatient‐based surveillance, the WHO ARI case definition was used at these sites.

**Table 1:** Site profile, WHO RSV surveillance, 2017‐19

Country	Duration of surveillance	No. of sentinel sites	Patients under surveillance	Number of clinical samples considered for analysis
Argentina	2017 Week 1‐2019 Week 14	6	Inpatient	1660
Australia	2017 Week 31‐ 2019 Week 17	1	Inpatient	2005
Brazil	2017 Week 3‐2019 Week 7	2	Inpatient + outpatient	756
Canada	2017 Week 44‐2018 Week 22; and 2018 Week 44‐2019 Week 22	12	Inpatient	4892
Chile	2017 Week 1‐2019 Week 13	2	Inpatient	1134
Côte d’Ivoire	2017 Week 1‐2019 Week 1	9	Inpatient + outpatient	3362
Egypt	2017 Week 1‐2019 Week 14	6	Inpatient	1688
India	2017 Week 1‐2019 Week 9	11	Inpatient + outpatient	1998
Mongolia	2017 Week 2‐2019 Week 15	7	Inpatient + outpatient	1803
Mozambique	2017 Week 1‐2019 Week 3	4	Inpatient + outpatient	1329
Russian Federation	2017 Week 1‐2019 Week 15	18	Inpatient	2067
South Africa	2017 Week 1‐2019 Week 18	5	Inpatient	6002
Thailand	2017 Week 1‐2019 Week 5	11	Inpatient + outpatient	3106
United Kingdom^a^	2017 Week 39‐2019 Week 12	70 GP	Outpatient	4250

Abbreviation: GP, General practitioners.

a Surveillance restricted to England only.

### 2.3 Sampling of cases

Argentina, Brazil, Chile, Egypt, the Russian Federation, and South Africa screened patients from all age groups admitted to sentinel hospitals, whereas Australia and Canada screened pediatric hospital admissions only. Côte d'Ivoire, India, Mongolia, Mozambique, and Thailand screened patients from all age groups in both sentinel hospitals and clinics. The United Kingdom screened pediatric patients attending sentinel general practitioner clinics in England (Table 1).

At all sentinel sites, physicians and nurses were trained to screen patients meeting the extended SARI case definition for hospital‐based surveillance and ARI for clinic surveillance. Patients were screened for eligibility all year‐round, except for Canada where the RSV pilot ran annually from the beginning of November (epidemiological week 44) through to the end of May (epidemiological week 22). Countries were required to test a minimum of 1000 samples annually for RSV (250 patients in each of four age groups—less than 6 month, 6 months to <5 years, 5 years to <65 years, and 65 years or over). If there were inadequate numbers of eligible patients for RSV surveillance in any age group, countries had the option to make up the shortfall by enrolling patients from the ongoing routine influenza surveillance where the SARI and ARI/ILI case definitions were being used or based on the patient load at the sentinel site. The sampling strategy varied across countries and ranged from screening of all eligible patients to screening of eligible patients on certain days of the week. While the required minimum number of samples per week was 20, some countries received more samples during the influenza season while others received less than the expected samples per week due to lesser number of case‐patients with respiratory illness. In addition, some countries enhanced laboratory testing during periods of increased proportion of RSV‐positive specimens.

### 2.4 Collection of clinical samples and detection of RSV

Clinical specimen collection, transport, and storage of clinical samples were done as per WHO guidelines. Oropharyngeal, nasal or nasopharyngeal respiratory specimens (according to age) were collected and transported in viral transport media to the National Influenza Centre or the national public health laboratory for testing for RSV and influenza virus.^11^ Before Initiating testing of clinical samples, all countries successfully participated in an external molecular quality assurance panel for the detection of RSV, which was developed by the United States Centers for Disease Control and Prevention (US CDC). Clinical specimens were batch tested in their respective countries. All laboratories used real‐time reverse transcription‐polymerase chain reaction (RT‐PCR) for RSV detection; while most laboratories used an assay developed by the US CDC, which was a monoplex RT‐PCR assay that detected the conserved regions of the RSV matrix protein gene. A few laboratories opted to continue using commercial or in‐house developed RT‐PCR assays after validation against the US CDC assay. Reagents were supplied by the US CDC through the influenza Reagent Resource (IRR; https://www.internationalreagentresource.org). Seven countries (Argentina, Australia, Canada, India, South Africa, Thailand, and the United Kingdom) used multiplex RT‐PCR to further subtype all or a representative sample of RSV‐positive specimens as RSV‐A and RSV‐B. All countries also tested specimens for influenza.

### 2.5 Duration of surveillance

During the pilot, RSV surveillance was conducted year‐round by all countries even though seasonality was already well‐established in some temperate countries, except for Canada where the RSV pilot ran within the timeframe of well‐established seasonality from the beginning of November (epidemiological week 44) through to the end of May (epidemiological week 22; Table 1).

### 2.6 Data collection and analysis

Data from surveillance sites were uploaded to the WHO's web‐based FluMart data platform by the respective countries. To analyze RSV seasonality, the date of specimen collection was used to aggregate by epidemiologic week (EW). When the date of specimen collection was missing, the date of onset of symptoms was used instead. Since date of onset was missing in 6560 cases and date of specimen collection was most definitive, it was used for construction of the epi curve. All specimens collected using the SARI, extended SARI and ARI/ILI case definition were pooled per country for the RSV seasonality graphs. Temporal plots of RSV and influenza activity were smoothed using the 3‐week moving average in R version 3.5.3 (R Foundation for Statistical Computing). Data points where only one specimen was tested in the week were removed from the analysis. Seven countries (Brazil, Chile, Egypt, India, Mozambique, South Africa, and the United Kingdom) described their seasons. For the remaining countries, seasons were derived from the World Meteorological Organization website or were taken from the official web site of the country.^12^

For analyzing seasonality, a threshold of 10% RSV positivity for two consecutive weeks was used to indicate the onset of the RSV season and similarly a percent positivity of <10% for two consecutive weeks was used to indicate the end of the season. For Canada, the number of RSV‐positive cases was used to analyze seasonality as the total number of specimens tested was not reported. In countries with multiple sentinel sites, data were pooled for each country's seasonality analysis. For countries which typed RSV cases (Argentina, Australia, Canada, India, Thailand, South Africa, and the UK), data were also analyzed by RSV subtypes.

## 3 RESULTS

Fourteen countries provided data for the seasonality analysis (Table 1). Nine of 14 countries initiated surveillance from EW 01 of 2017 through a variable period in 2019 (EW 01‐25) (Table 1). Data for two RSV seasons were captured across all countries except for Côte d'Ivoire. A total of 245 of 33 583 samples (0.73%) had both date of specimen collection and onset of symptom missing and were excluded from analysis.

**Figure 2:**
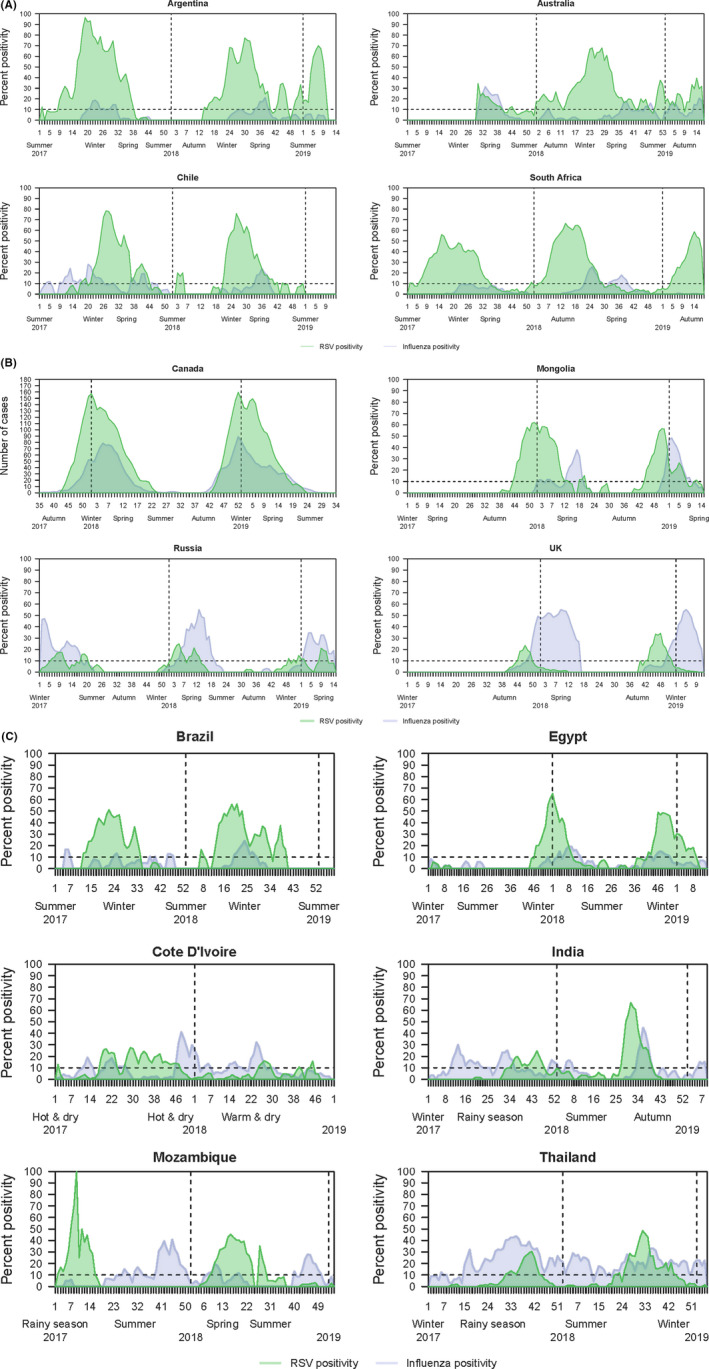
A, RSV and influenza seasonality of countries with temperate climate located in the southern hemisphere. B, RSV and influenza seasonality of countries with temperate climate located in the Northern hemisphere. C, RSV and influenza seasonality of countries with subtropical/tropical climate

The percentages of specimens testing positive for RSV and influenza during each epidemiological week of surveillance are shown in Figure 2. In several temperate countries (Argentina, Australia, Chile, Canada, Mongolia, Russian Federation, and the UK), RSV activity tended to be greatest in the cooler months of the year, but there was substantial variability in timing between countries, and some year‐to‐year variation within countries. As shown in Figure 2A, the RSV activity in Argentina in 2017/2018 began in early autumn and peaked in winter. However, in 2018/19 RSV activity continued in the spring and summer seasons (RSV season duration 2017/18 = 28 weeks; 2018/19 = 50 weeks). In Australia, there was some RSV activity in the summer months, but it peaked in the winter (RSV season duration; 2018/19 = 52 weeks). In Chile, the RSV season started in late autumn‐early winter in both years and ended in spring (RSV season duration 2017 = 22 weeks; 2018 = 18 weeks). In South Africa, RSV activity started in late summer in each of the 3 years of surveillance, peaked in autumn/winter, and ended in spring (RSV season duration 2017 = 28 weeks; 2018 = 27 weeks).

In Canada, no RSV surveillance was conducted in the summer; RSV activity started in the late autumn and continued through to spring, with peak activity during winter (RSV season duration 2017/18 = 17 weeks; 2018/19 = 16 weeks). In Mongolia, RSV activity started in early winter and ended by spring (RSV season duration 2017/18 = 10 weeks; 2018/19 = 17 weeks). In Russia, relatively smaller numbers of specimens tested positive for RSV, compared to most other countries, with highest RSV activity in winter and spring (RSV season duration 2017/18 = 7 weeks; 2018/19 = 10 weeks). The UK also had relatively low RSV positivity rates, with activity starting in mid‐autumn and ending by mid‐winter (RSV season duration 2017/18 = 7 weeks; 2018/19 = 10 weeks). (Figure 2B).

Six countries had a subtropical/tropical climate (Figure 2C). In Brazil, in both years of surveillance RSV activity started in early autumn and finished toward the end of winter (RSV season duration 2017 = 21 weeks; 2018 = 27 weeks). In Egypt, RSV activity started in early winter and finished in early spring (RSV season duration 2017/18 = 17 weeks; 2018/19 = 18 weeks).

In Côte d'Ivoire, seasonality was less clear, but most RSV activity was detected toward the end of the hot and dry season, through to the hot and wet season and declined at the beginning of the warm and dry season (RSV season duration 2017 = 30 weeks). In India, activity began in the rainy season and continued to the beginning of winter, though the patterns in 2017 and 2018 differed (RSV season duration 2017 = 13 weeks; 2018 = 12 weeks). In Mozambique, RSV activity started toward the end of the rainy season and finished in late spring or early winter (RSV season duration 2017 = 10 weeks; 2018 = 17 weeks). Thailand had RSV activity starting in the early part of the rainy season and ended toward the end of the rainy season in the first year with activity beginning earlier in the second year of surveillance (RSV season duration 2017 = 6 weeks; 2018 = 20 weeks).

Respiratory syncytial virus seasonality when compared with that of influenza showed different patterns across various countries. Influenza and RSV activity peaks were observed during the same period in some countries (eg, Australia, Brazil, Canada, Egypt, and Russia,). The RSV season preceded the influenza season by 6‐9 weeks in Argentina, Mongolia South Africa, and the UK. In counties, where the RSV season preceded the influenza season, the end of RSV activity varied. In Argentina and South Africa, which were both temperate regions, RSV season ended with the start of the influenza season. In the UK, the RSV season ended before influenza activity peaked. In Mozambique, the 2017 RSV season ended before influenza activity commenced while in 2018, both RSV and influenza activity overlapped but had different peaks. However, influenza was not observed after week 16 and by week 20, a downward trend was observed in RSV activity. Year‐round baseline activity of influenza was observed in Côte d'Ivoire, India, and Thailand but RSV was distinctly heightened during the rainy season. In 2017, India observed influenza year‐round and RSV was predominantly observed during the rainy season and continuing through the winter of 2017‐18 and summer of 2018. In 2018 in India, influenza was not observed during summer but was circulated in the rainy season. Although there was some similarity with respect to the timings of the periods of high positivity for RSV and influenza, there was considerable variation among the participating countries.

Among the pilot countries that performed RSV sub‐typing, in 2017, RSV‐A circulation predominated in Argentina (91% of 237 RSV positives) and the UK (62.5% of 104), whereas RSV‐B predominated in Australia (75% of 101), India (98% of 54), South Africa (64.4% of 605), and Thailand (57% of 148). In the 2018 season, RSV‐B was the predominantly circulating type in all countries: Argentina (93% of 215), Australia (54% of 326), India (100% of 85), Thailand (61% of 111), and the UK (73% of 33), except South Africa where RSV‐A predominated (61% of 727). In Canada, RSV‐A and RSV‐B co‐circulated (50% RSV of 105) predominantly RSV‐B (66% of 64) at the western sentinel and RSV‐A (76% of 41) at the eastern sentinel. Predominance of subtype did not affect the timing of RSV activity peaks in any of the countries.

## 4 DISCUSSION

We describe the RSV seasonality in fourteen countries participating in the WHO RSV surveillance. This is the first time the GISRS platform has included additional pathogen surveillance using a broader case definition and which allowed standardization of RSV surveillance protocols and facilitated the description of RSV seasonality in participating countries. Intervention strategies for RSV may need to account for the seasonal nature of RSV, as the protection conferred by monoclonal antibodies or maternal immunization may be short lasting.^13^ Knowledge of the local seasonality patterns of RSV will help target intervention strategies for RSV, saving costs and allowing maximum benefit.

Distinct annual peaks of RSV detection were observed across all the study sites. The magnitude of the peak and the duration of increased activity varied across countries, and there was some year‐to‐year variation within countries. RSV activity observed among countries may be influenced by weather pattern.[14, 15, 16, 17] While it was possible to compare/group some countries, there was still considerable variation even among countries with similar climate zones. In the temperate regions in both hemispheres, the main RSV season period occurred during the colder months (Argentina, Australia, Canada, Chile, Mongolia, Russian Federation, and the UK). Associations between low temperatures and widespread RSV circulation in temperate climates have been well described.^17^, ^18^, ^19^, ^20^, ^21^ Although South Africa lies in the temperate zone, RSV activity varied; RSV peak was observed in autumn of 2017 and 2018, which was not the coldest period of the year. This temperature‐dependent pattern appears to be independent of precipitation patterns.^22^ In subtropical countries with lower annual temperatures like Brazil and Egypt, RSV peaks occurred in the cooler months of the year. These findings are consistent with previous studies.^17^, ^18^ In the tropics, while RSV peaks were observed primarily during rainy seasons (Côte d'Ivoire, India, Mozambique, and Thailand), residual viral activity was seen throughout the year. Other studies in tropical areas have reported RSV seasons occurring during periods of high precipitation.^23^, ^24^ Similarly, earlier studies from Calcutta and The Gambia have reported outbreaks mainly during rainy season.^25^, ^26^, ^27^ This could support the hypothesis that due to increased precipitation there is more indoor crowding, facilitating RSV transmission in the tropics.^28^

The timing of the RSV season was not dependent on subtype (type A or type B) that occurred, or the proportions of A/B seen in that season in countries that performed this typing. This aspect will be further studied in the next phase of the WHO RSV surveillance program.

Respiratory syncytial virus had broader distribution of peak timings, relative to that of influenza, even within the temperate zone. Although most countries in our study experienced distinct respiratory virus seasons, it is noteworthy that there was a bi‐annual influenza peak in Chile in 2017. Although distinct RSV seasonality was observed in tropical countries, that is, Brazil, Côte d'Ivoire, India, Mozambique, and Thailand, Influenza activity was observed year‐round, which is consistent with previous reports.^29^, ^30^ This could influence the country‐specific RSV vaccination strategies and timing in relation to influenza vaccination. However, additional years of surveillance data are required for better understanding of the variability in the distribution of RSV timing relative to influenza and whether RSV–A and RSV‐B subtypes have any role to play in multi‐year periodicity of RSV compared to Influenza.

There are several limitations to be noted. First, the duration of surveillance varied between countries and covered only two seasons. It is not possible to establish reliably the consistency of seasonal patterns of RSV from year‐to‐year in these countries. Second, we did not consider detailed meteorological data for each country during the actual period of surveillance and, as such, seasons were used descriptively for our assessment based on usual patterns. Third, RSV activity observed was limited to the catchment areas of participating surveillance sites within the countries and may not be representative of the whole country, especially in countries with a large latitudinal spread with multiple climate zones. Some of these limitations will be addressed in the second phase of global RSV surveillance initiative. Surveillance needs to be conducted over a longer period, using uniform case definition and sampling strategies and ensuring geographical representativeness in larger countries. It would also be useful to evaluate the threshold of 10% RSV positivity to describe onset and offset of the seasonal RSV epidemic. With more data available other methods of analyzing seasonal data may be used.^16^ Despite its limitations, this pilot provides a better understanding of the seasonality of RSV circulation globally. Additional years of RSV surveillance data from different geographical regions within the same country will provide more robust RSV seasonality patterns.

In conclusion, we observed distinct annual peak activity of RSV in different regions of the world. Timing of RSV epidemics varied among participating countries, and it generally overlapped with seasonal influenza epidemics in most countries. The GISRS platform can be leveraged for generating standardized and quality data on RSV circulation. These data will be useful for deciding the best time for targeting interventions such as future RSV vaccination and the use of monoclonal antibodies.

## DISCLAIMER

The authors are responsible for the views expressed in this publication, and they do not necessarily represent the decisions, policy, or views of the World Health Organization or their Institutes.

## APPENDIX 1

WHO RSV Surveillance Group: Amal Barakat (Eastern Mediterranean Region Office, World Health Organization, Cairo, Egypt), Sumit Bhardwaj (National Institute of Virology, Indian Council of Medical Research, Pune, India), Shobha Broor (Medicine and Health Sciences, Shree Guru Gobind Singh Tricentenaryl University, Gurugram, India), Alyeksandr Burmaa (National Influenza Surveillance Division, National Center for Communicable Diseases, Ministry of Health, Ulaanbaator, Mongolia), Harry Campbell (Usher Institute of Population Health Research and Informatics, University of Edinburgh, Edinburgh, United Kingdom), Daouda Coulibaly (Epidemiological Surveillance and Research, Institut National de l'Hygiène Publique, Abidjan, Côte d'Ivoire), Nigel Crawford (Royal Children's Hospital, Melbourne, Australia), Manal Fahim (Department of Surveillance and Epidemiology, Ministry of Health and Population, Cairo, Egypt), Belinda Herring (African Region Office, World Health Organization, Brazzaville, Republic of Congo), Harish Nair (Usher Institute of Population Health Research and Informatics, University of Edinburgh, Edinburgh, United Kingdom), Rakhee Palekar (Pan American Health Organization, Washington DC, United States), Richard Pebody (Flu Surveillance, Public Health England, London, United Kingdom), Soatiana Rajatonirina (African Region Office, World Health Organization, Brazzaville, Republic of Congo), Marilda Siqueira (Institute Oswaldo Cruz/FIOCRUZ, Rio de Janerio, Brazil), Peter G. Smith (MRC Tropical Epidemiology Group, London School of Hygiene and Tropical Medicine, London, United Kingdom), Elizaveta Smorodintseva (Laboratory for studying risk factors of Influenza and ARVI, Smorodintsev Research Institute of Influenza, Ministry of Health of the Russian Federation, St. Petersburg, Russian Federation), Viviana Sotomayor (Division de Planificacion Sanitaria, Ministerio de Salud Chile, Santiago, Chile), Florette Treurnicht (Centre for Respiratory Diseases and Meningitis, National Institute for Communicable Diseases, Johannesburg, South Africa), Miquelina Vaz (Laboratório de Isolamento Viral, Instituto Nacional de Saúde, Maputo, Mozambique), Marietjie Venter (Center for Viral Zoonosis, Department of Medical Virology, University of Pretoria, South Africa), Niteen Wairagkar (Bill and Melinda Gates Foundation, Seattle, United States), Maria Zambon (Virus Reference Department, Public Health England, London, United Kingdom).

## References

[1] Collins PL , Chanock RM , Murphy BR . Respiratory syncytial virus In: Knipe DM, Howley PM, eds. Fields virology. Philadelphia: Lippincott‐Raven; 2001:1443‐1485.

[2] Shi T , McAllister DA , O'Brien KL , et al. Global, regional and national disease burden estimates of acute lower respiratory infections due to respiratory syncytial virus in young children in 2015: a systematic review and modelling study. Lancet. 2017;390:946‐958. 28689664 10.1016/S0140-6736(17)30938-8 PMC5592248

[3] Saha SK , Schrag SJ , El Arifeen S , et al. Causes and incidence of community‐ acquired serious infections among young children in south Asia (ANISA): an observational cohort study. Lancet. 2018;392(10142):145‐159. 30025808 10.1016/S0140-6736(18)31127-9 PMC6053599

[4] Fleming DM , Taylor RJ , Lustig RL , et al. Modeling estimates of the burden of respiratory syncytial virus infection in adults and the elderly in the United Kingdom. BMC Infect Dis. 2015;15:443. 26497750 10.1186/s12879-015-1218-z PMC4618996

[5] Respiratory syncytial virus. https://www.cdc.gov/rsv/clinical/index.html. Accessed August 29, 2018.

[6] Han LL , Alexander JP , Anderson LJ . Respiratory syncytial virus pneumonia among the elderly: an assessment of disease burden. J Infect Dis. 1999;179(1):25‐30. 9841818 10.1086/314567

[7] Belongia EA , King JP , Kieke BA , et al. Clinical features, severity, and incidence of RSV illness during 12 consecutive seasons in a community cohort of adults ≥60 years old. Open Forum Infect Dis. 2018;5(12). https://doi.org/https://doi.org/10.1093/ofid/ofy316 10.1093/ofid/ofy316 PMC6306566 30619907

[8] Mazur NI , Higgins D , Nunes MC , et al. The respiratory syncytial virus vaccine landscape: lessons from the graveyard and promising candidates. Lancet Infect Dis. 2018;18(10):e295‐e311. 29914800 10.1016/S1473-3099(18)30292-5

[9] Pablo OP , Antonio JG , Irene RC , et al. Respiratory syncytial virus seasonality: a global overview. J Infect Dis. 2018;217(9):1356‐1364. 29390105 10.1093/infdis/jiy056

[10] WHO strategy to pilot global respiratory syncytial virus surveillance based on the global influenza surveillance and response system (GISRS). https://apps.who.int/iris/handle/10665/259853. Accessed March 7^th^, 2019.

[11] WHO . Guidelines for the collection of clinical specimens during field investigation of outbreaks. https://apps.who.int/iris/bitstream/handle/10665/66348/WHO_CDS_CSR_EDC_2000.4.pdf;jsessionid=183CE24D75CC2A139A922D5CC7567233?sequence=1. Accessed August 12^th^, 2018.

[12] Durham immigration portal . Weather –four season. https://www.durhamimmigration.ca/en/moving-to-durham-region/weather–-four-seasons.aspx. Accessed October 10, 2018.

[13] Aranda SS , Polack FP . Prevention of pediatric respiratory syncytial virus lower respiratory tract illness: perspectives for the next decade. Front Immunol. 2019;10:1006. 31134078 10.3389/fimmu.2019.01006 PMC6524688

[14] Haynes AK , Manangan AP , Iwane MK , et al. Respiratory syncytial virus circulation in seven countries with global disease detection regional centers. J Infect Dis. 2013;208(Suppl 3):S246‐S254. 24265484 10.1093/infdis/jit515

[15] Bloom‐Feshbach K , Alonso WJ , Charu V , et al. Latitudinal variations in seasonal activity of influenza and respiratory syncytial virus (RSV): a global comparative review. PLoS One. 2013;8(2):e54445. 23457451 10.1371/journal.pone.0054445 PMC3573019

[16] Kamigaki T , Chaw L , Tan AG , et al. Seasonality of influenza and respiratory syncytial viruses and the effect of climate factors in subtropical‐tropical Asia using influenza like Illness surveillance data, 2010–2012. PLoS One. 2016;11(12):e0167712. 28002419 10.1371/journal.pone.0167712 PMC5176282

[17] Li Y , Reeves RM , Wang X , et al. Global patterns in monthly activity of influenza virus, respiratory syncytial virus, parainfluenza virus, and metapneumovirus: a systematic analysis. Lancet Glob Health. 2019;7(8):e1031‐e1045. 31303294 10.1016/S2214-109X(19)30264-5

[18] Sloan C , Moore ML , Hartert T . Impact of pollution, climate, and sociodemographic factors on spatiotemporal dynamics of seasonal respiratory viruses. Clin Transl Sci. 2011;4:48‐54. 21348956 10.1111/j.1752-8062.2010.00257.x PMC3071158

[19] Robertson SE , Roca A , Alonso P , et al. Respiratory syncytial virus infection: denominator‐based studies in Indonesia, Mozambique, Nigeria and South Africa. BullWorld Health Organ. 2004;82:914‐922. PMC2623097 15654405

[20] Hervas D , Reina J , Hervas JA . Meteorologic conditions and respiratory syncytial virus activity. Pediatr Infect Dis J. 2012;31:e176‐e181. 22572747 10.1097/INF.0b013e31825cef14

[21] Martin AJ , Gardner PS , McQuillin J . Epidemiology of respiratory viral infection among paediatric inpatients over a six year period in north‐east England. Lancet. 1978;2:1035‐1038. 82045 10.1016/s0140-6736(78)92351-6

[22] Gilchrist S , Torok TJ , Gary HE Jr , Alexander JP , Anderson LJ . National surveillance for respiratory syncytial virus, United States 1985–90. J Infect Dis. 1994;170:986‐990. 7930745 10.1093/infdis/170.4.986

[23] Joosting AC , Harwin RM , Orchard M , Martin E , Gear JH . Respiratory viruses in hospital patients on the Witwatersrand. A 7‐ year study. S Afr Med J. 1979;55:403‐408. 219551

[24] Khor CS , Sam IC , Hooi PS , Quek KF , Chan YF . Epidemiology and seasonality of respiratory viral infections in hospitalized children in Kuala Lumpur, Malaysia: a retrospective study of 27 years. BMC Pediatr. 2012;12:32. 22429933 10.1186/1471-2431-12-32 PMC3337250

[25] Fry AM , Chittaganpitch M , Baggett HC , et al. The burden of hospitalized lower respiratory tract infection due to respiratory syncytial virus in rural Thailand. PLoS One. 2010;5:e15098. 21152047 10.1371/journal.pone.0015098 PMC2994907

[26] Kloene W , Bang FB , Chakraborty SM , et al. A two‐year respiratory virus survey in four villages in West Bengal, India. Am J Epidemiol. 1970;92:307‐320. 4319714 10.1093/oxfordjournals.aje.a121212

[27] Hillis WD , Cooper MR , Bang FB , Dey AK , Shah KV . Respiratory syncytial virus infection in children in West Bengal. Indian J Med Res. 1971;59:1354‐1364. 5161563

[28] Ota WK , Bang FB . A continuous study of viruses in the respiratory tract in families of a Calcutta bustee. Am J Epidemiol. 1972;95:371‐383. 4335609 10.1093/oxfordjournals.aje.a121406

[29] Genee SS , Kyle PM , James LC , Timothy JW , Cynthia JL , Elizabeth DH . Extreme precipitation and emergency room visits for influenza in Massachusetts: a case‐crossover analysis. Environ Health. 2017;16:108. 29041975 10.1186/s12940-017-0312-7 PMC5645981

[30] Weber MW , Dackour R , Usen S , et al. The clinical spectrum of RSV disease in The Gambia. Pediatr Infect Dis J. 1997;17:224‐230. 10.1097/00006454-199803000-00010 9535250

[31] Hirve S , Newman LP , Paget J , et al. Influenza seasonality in the tropics and subtropics ‐ when to vaccinate? PLoS One. 2016;11(4). 10.1371/journal.pone.0153003 PMC4847850 27119988

